# Unguarded liabilities: *Borrelia burgdorferi’s* complex amino acid dependence exposes unique avenues of inhibition

**DOI:** 10.3389/frabi.2024.1395425

**Published:** 2024-05-20

**Authors:** Katrina J. Holly, Arti Kataria, Daniel P. Flaherty, Ashley M. Groshong

**Affiliations:** ^1^ Borch Department of Medicinal Chemistry and Molecular Pharmacology, College of Pharmacy, Purdue University, West Lafayette, IN, United States; ^2^ Laboratory of Bacteriology, Rocky Mountain Laboratories, Division of Intramural Research, National Institute of Allergy and Infectious Diseases, National Institutes of Health, Hamilton, MT, United States

**Keywords:** *Borrelia burgdorferi*, Lyme disease, spirochete, oligopeptide, antibiotic

## Abstract

Recent reports from the Centers for Disease Control and Prevention approximate 500,000 cases of Lyme disease in the United States yearly, a significant economic burden on the healthcare system. The standard treatment for Lyme disease includes broad-spectrum antibiotics, which may be administered for extensive periods of time and result in significant impacts to the patient. Recently, we demonstrated that *Borrelia burgdorferi*, the causative agent of Lyme disease, is uniquely dependent upon peptide acquisition via an oligopeptide transport (Opp) system. This dependence appears unique to the spirochete; thus, the Opp system may constitute a novel and specific target for the inhibition of *B. burgdorferi*. For proof of concept, we conducted a pilot screen to determine if the Opp system constitutes a viable inhibitor target. OppA2 was utilized as our target protein as it is the most prolific peptide-binding protein throughout the enzootic cycle. We validated a thermal shift assay (TSA) to detect ligand binding against OppA2 and performed a high-throughput screen of 2,240 molecules from a diversity set library. The TSA results identified eight compounds (C1–8) demonstrating potential binding to OppA2, and growth assays identified C2 and C7 as inhibitors of *B. burgdorferi* growth. We confirmed by TSA that these two compounds interact with additional *B. burgdorferi* OppAs, potentially resulting in a cumulative inhibitory effect. Additionally, we showed that these compounds have no effect on *Escherichia coli*, a bacterium that encodes a dispensable Opp system which serves only as an ancillary nutrient transporter. These data demonstrate that the Opp system of *B. burgdorferi* acts as a viable drug target, with the potential for targeting multiple OppAs with a single compound. Moreover, the lack of inhibition against *E. coli* suggests that selective targeting of *B. burgdorferi* via the Opp system may be possible.

## 1 Introduction

The causative agent of Lyme disease, *Borrelia burgdorferi*, was discovered in the early 1980s (Burgdorfer et al., 1982). Since then, Lyme disease has emerged as the most prevalent vector-borne illness in the United States (Nelson et al., 2015) and continues to undergo geographic expansion as global climate change expands the arthropod vector’s geographical distribution (Ostfeld and Brunner, 2015). Standard treatments for Lyme disease include antibiotics such as amoxicillin, doxycycline, and ceftriaxone; however, these treatment regimens are often months-long and may include daily intravenous administration (Steere et al., 2016). Since these antibiotic courses do not uniquely target *B. burgdorferi*, patients contend with ablation of their microbiome and other off-target effects during and after treatment. Furthermore, a subset of patients that receive treatment suffer long-term debilitation from persistent symptoms, a condition termed Post-treatment Lyme Disease Syndrome (PTLDS) (Aucott et al., 2022). Currently, it is unclear what drives the persistent symptoms during PTLDS, though possibilities include persistent bacteria, residual immunoreactive protein, autoimmune response, protracted immune dysregulation, or long-lasting off-target effects of the antibiotic treatments (Cabello et al., 2007; Hodzic et al., 2008; Marques, 2008; Bockenstedt et al., 2012; Cabello et al., 2017; Crossland et al., 2018; Bobe et al., 2021; Verschoor et al., 2022).

*B. burgdorferi* is a spirochetal diderm with a reduced and fragmented genome consisting of a single linear chromosome and approximately 20 circular and linear plasmids (Fraser et al., 1997; Casjens et al., 2000). The spirochete is an extreme auxotroph, lacking a significant number of synthetic pathways, and must scavenge a large array of nutrients from both its arthropod vector (*Ixodes scapularis*) and host environments (Fraser et al., 1997). While *B. burgdorferi*’s requirement for many substrates (e.g., carbohydrates and fatty acids) have been well studied, only recently was it demonstrated that the spirochete requires peptide uptake for viability (Groshong et al., 2017), a feature that appears unique among bacterial species to date. The peptide transport system relies upon five oligopeptide-binding proteins (OppA1–5) to aid in the transport of peptides across the bacterial inner membrane, along with two heterodimeric permeases (OppB1C1 and OppB2C2) and a single nucleotide-binding domain-containing heterodimer (OppDF) to hydrolyze ATP during peptide translocation (Fraser et al., 1997; Casjens et al., 2000; Groshong et al., 2017). Once in the cytoplasm, the peptides can be disassembled and utilized to build bacterial proteins and to drive other potential physiological functions such as osmotolerance and cell signaling (Monnet, 2003). Unexpectedly, our group found that ablation of this system in *B. burgdorferi* and the subsequent amino acid starvation caused a significant maladaptative response, resulting in cell elongation, flagellar dysregulation, and, ultimately, cell death (Groshong et al., 2017). These phenotypes contrasted with the typical bacterial responses to amino acid starvation which generally triggers a bacterial stringent response resulting in reduced doubling time and shortened cell bodies (Grossman et al., 1982; Wang et al., 2020). To date, *B. burgdorferi* is the only bacterium that has demonstrated dependence on peptide uptake for basic viability.

In other bacterial species, the Opp system serves to scavenge peptides as an alternative to amino acid synthesis and is utilized as an ancillary nutrient acquisition system when peptides are available (Monnet, 2003). OppAs have been shown to have a highly conserved structure with two globular domains connected by a hinge region (Monnet, 2003). Unlike other binding proteins in bacteria which display selectivity for ligands via ligand binding residue interactions with specific functional groups, residues in the peptide binding pocket interact with ligands via the peptide backbone in a non-specific manner, resulting in highly promiscuous binding (Klepsch et al., 2011). This feature allows an OppA to transport a diverse array of peptides, meeting a wide range of amino acid needs. Conversely, some bacterial OppA homologs are highly specific for certain peptide derivatives. One example is the *Escherichia coli* MppA which selectively binds the murein tripeptide fragment formed during peptidoglycan recycling (Park et al., 1998). *B. burgdorferi* encodes for five OppAs, which are uniquely regulated throughout the enzootic cycle (Groshong et al., 2021b). Previous studies strongly suggest that each OppA transports peptides (Lin et al., 2001; Wang et al., 2004); however, modeling showed variations within the ligand binding sites that suggest each OppA may display unique or overlapping preference for different peptides (Groshong et al., 2017). Loss of individual OppAs result in unique phenotypes, supporting the hypothesis that the different OppAs are not wholly redundant but rather that each perform compartmentalized functions (Groshong et al., 2021b). Among the five OppAs, we theorize that OppA2 is critical to the spirochete because it is the only OppA conserved among all species of *Borrelia* (Groshong et al., 2017); it is the most highly expressed OppA throughout the enzootic cycle, and its loss inhibits hematogenous dissemination during mammalian infection (Groshong et al., 2021b).

Due to the essential nature of the Opp system in *B. burgdorferi*, we endeavored to test its potential as an unexplored target for bacterial inhibition. While the loss of a single OppA in *B. burgdorferi* does not affect viability, the highly conserved structure of the OppAs suggests that a single inhibitor may target more than one OppA. Herein we utilized OppA2 as a target to screen for prospective inhibitors via a high-throughput binding assay. We developed and validated a thermal shift assay (TSA) to identify hit compounds that bind OppA2, followed by the biological evaluation of hits in bacterial growth assays. From a pilot screen of 2,240 compounds, we identified eight which bind OppA2, and, ultimately, two compounds that display a significant inhibition of *B. burgdorferi* growth. This study demonstrates that the previously unexplored Opp system components may be targets for therapeutic intervention in *B. burgdorferi* infection and further suggests that compounds targeting the Opp system may be selective for *B. burgdorferi*.

## 2 Materials and methods

### 2.1 Bacterial strains and culture conditions

All strains used in this study are listed in **Table 1**. *Escherichia coli* strains were grown in Luria–Bertani (LB) broth or LB plates with appropriate antibiotics [kanamycin (Kan; 50 μg/mL)] at 37°C unless otherwise noted. All *Borrelia burgdorferi* strains used in this study were grown in modified Barbour–Stoenner–Kelly-II (BSK-II) medium (Pollack et al., 1993) supplemented with 6% rabbit serum and appropriate antibiotics [kanamycin (Kan; 400 μg/mL), gentamycin (Gent; 50 μg/mL)] at 37°C and 5% CO_2_.

**Table 1:** Bacterial strains and plasmids used in this study.

Strain/plasmid	Description	Antibiotic resistance	Reference
*B. burgdorferi*			
BbG101	Wild-type strain B31 5A4 (*wt*)	–	(Purser and Norris, 2000)
BbG117	B31 5A18 NP1 *bb0329tn* (*oppA2tn*)	Kan/Gent	(Groshong et al., 2021b)
*E. coli*			
Top10	F^-^ *mcr*A Δ(*mrr*-*hsd*RMS-*mcr*BC) φ80*lac*ZΔM15 Δl*ac*X74 *rec*A1 *ara*D139 Δ(*ara*-*leu*)7697 *gal*U *gal*K *rps*L (Str^R^) *end*A1 *nup*G	–	Invitrogen
Stellar	F^–^, *endA1*, *supE44*, *thi-1*, *recA1*, *relA1*, *gyrA96*, *phoA*, *Φ80d lacZΔ M15*, *Δ*(*lacZYA-argF*) *U169*, *Δ*(*mrr-hsdRMSmcrBC*), *ΔmcrA*, *λ* ^–^	–	Takara Bio
Plasmids			
pET28a	pET expression vector for 6his-tagged protein expression	Kan	Novagen
pG225	pET28a-OppA1opt-SS	Kan	This study
pG226	pET28a-OppA2opt-SS	Kan	This study
pG227	pET28a-OppA3opt-SS	Kan	This study
pG228	pET28a-OppA4opt-SS	Kan	This study
pG229	pET28a-OppA5opt-SS	Kan	This study

### 2.2 Generation of expression constructs

All primers and plasmids used in this study are listed in **Table 2**. *Borrelia burgdorferi* strain B31 OppA genes [OppA1 (BB0328), OppA2 (BB0329), OppA3 (BB0330), OppA4 (BBB16), and OppA5 (BBA34)] were codon-adapted for expression in *E. coli*, synthesized, and cloned into pUCIDT-Kan (IDT, USA). The lipoprotein signal sequence for each gene was identified for each protein using LipoP (Juncker et al., 2003) and excluded during expression cloning. Each codon-optimized open reading frame (ORF) was amplified using CloneAmp HiFi Premix and the primers listed in **Table 2**. Gel-purified fragments and Novagen’s pET28a (Millipore, USA) linearized with NdeI/XhoI were joined using the InFusion HD cloning kit (Takara Bio, USA) and selected on Kan plates. Individual clones were confirmed by Sanger sequencing.

**Table 2:** Oligonucleotide primers used in this study.

Designation	Sequence (5′–3′)	Purpose	Reference
5′-SS oppA1opt28a	**GCGC*GGCAGCC*ATATG**TGTATTTCTAACGCTAAAAAAGAAAAAATTGTGTTTC	OppA1 expression	This study
3′oppA1opt28a	**GGTGGTGGTG*CTCGAG*** TTACTTCTTCGTTTTAATATCTTCGTACAAATAGC	OppA1 expression	This study
5′-SS oppA2opt28a	**GCGC*GGCAGCC*ATATG**TGCAACAACAAAGAGCGCAAAGAA	OppA2 expression	This study
3′oppA2opt28a	**GGTGGTGGTG*CTCGAG*** TTACTTGTTCTTTAACTTCAACTGACTAAGATC	OppA2 expression	This study
5′-SS oppA3opt28a	**GCGC*GGCAGCC*ATATG**TGTAACAATAACAGTGAGAAAGAGAAATTGGC	OppA3 expression	This study
3′oppA3opt28a	**GGTGGTGGTG*CTCGAG*** TTAATTGTGTTTGGCGTTCTTAATTGG	OppA3 expression	This study
5′-SS oppA4opt28a	**GCGC*GGCAGCC*ATATG**TGCGTGAACGAGAGTAACCGTAATAAG	OppA4 expression	This study
3′oppA4opt28a	**GGTGGTGGTG*CTCGAG*** TCACTTGATTGGTTTAATCTCCGACAG	OppA4 expression	This study
5′-SS oppA5opt28a	**GCGC*GGCAGCC*ATATG**TGCTCGGCGATGAGTAAGCCTAA	OppA5 expression	This study
3′oppA5opt28a	**GGTGGTGGTG*CTCGAG*** TTATTCCTCAATCGGCTTAATCTCGC	OppA5 expression	This study
T7 Promoter	TAATACGACTCACTATAGGGGA	Sequencing	This study
T7 Terminator	ATCCGGATATAGTTCCTCCTTTCAGC	Sequencing	This study

Bold emphasis denotes overlap sequence for InFusion cloning. Italics emphasis denotes restriction sites.

### 2.3 Expression and purification of recombinant OppAs

Recombinant OppAs were expressed using standard auto-induction protocols as previously described (Studier, 2005). Briefly, *E. coli* clones containing expression constructs were grown in 5 mL LB–Kan overnight, and a secondary culture was passed into ZY media with Kan for auto-induction. Cultures were grown at 37°C, 220 rpm for 6 to 7 h and then shifted to 18°C overnight. Cells were harvested by centrifugation at 3,000 × *g* for 20 mins at 4°C. The cell pellet was lysed using BugBuster Protein extraction reagent (Millipore, USA) according to the manufacturer’s protocol. Lysates were centrifuged at 16,000 × *g* for 20 min at 4°C, and the supernatant was purified using a HisTrap HP column (Cytiva, USA) on AKTA Start (Cytiva, USA) by affinity chromatography. Purified protein was eluted in Tris buffer (50 mM Tris, 500 mM NaCl, 500 mM Imidazole, and 5% glycerol, pH 8.0), and protein fractions were evaluated by SDS-PAGE and visualized with Coomassie stain. To avoid dimer formation due to the presence of N-terminal cysteines, the affinity-purified samples were treated with 1 mM DTT prior to gel filtration chromatography. The affinity-purified fractions were pooled and concentrated and further purified on a Superdex 200 10/300 column (Cytiva, USA) using AKTA Pure chromatography system (Cytiva, USA) and size exclusion chromatography (SEC) buffer (10 mM Tris and 50 mM NaCl, pH 8.0). Secondary structure elements were confirmed using microfluidic modulation spectroscopy (MMS) with 1 mL of 2 mg/mL soluble protein on a Redshift AQS Pro with Apollo upgrades (RedshiftBio, USA).

### 2.4 Thermal shift assay for tripeptide binding

Initial thermal shift assay (TSA) conditions were modified from Jones et al. (2014). The assay buffer utilized for TSA was 1× PBS, pH 7.4. The Protein Thermal Shift™ Dye Kit (Thermo Fisher Scientific, USA) was used according to the manufacturer’s protocol. Peptides for *B. burgdorferi* OppA binding as determined by Lin et al. (2001) were generated along with additional test peptides (AAPPTec, USA) and used to optimize protein and peptide concentrations. Moreover, 0.1 mg/mL protein and 1 mM peptide were prepared in assay buffer. A total reaction volume of 20 μL was loaded in triplicate in MicroAmp™ Optical 96-Well Reaction Plate (Thermo Fisher Scientific, USA) sealed with MicroAmp™ Optical Adhesive Film (Thermo Fisher Scientific, USA) and run on Quantstudio 5 (Thermo Fisher Scientific, USA) according to the manufacturer’s protocol, and data was analyzed using Protein Thermal Shift™ software version 1.3 (Thermo Fisher Scientific, USA). The *T*_m_ for each sample was calculated from a Boltzmann fit to the melt curve.

### 2.5 Thermal shift assay optimization for inhibitor screening

The following was carried out to determine ideal TSA concentrations for all OppA proteins. The assay buffer utilized for TSA was 1× PBS, pH 7.4. Eight 10× OppA solutions ranging from 0.025 to 1 mg/mL were prepared in assay buffer, and an 8× dye solution from the Protein Thermal Shift™ Dye Kit was also prepared in assay buffer. Applied Biosystems MicroAmp^®^ EnduraPlate Optical 384-well reaction plates (Thermo Fisher Scientific, USA) were utilized for all TSAs. To each well was added 2 μL of respective 10× OppA solution, 2.5 μL of 8× dye, and 15.5 μL assay buffer, for a total volume of 20 μL and a final OppA2 concentration range of 0.0025 to 0.1 mg/mL in the assay. A negative control was also prepared, which contained only dye in assay buffer. Four replicates of each condition were run in the assay plate. The plate was sealed with MicroAmp™ Optical Adhesive Film and centrifuged. The TSA was run using an Applied Biosystems Viia 7 Real-Time PCR System according to the Protein Thermal Shift™ Dye Kit manufacturer’s protocol and analyzed using Protein Thermal Shift™ Software version 1.3. The *T*_m_ for each sample was calculated from a Boltzmann fit to the melt curve. The lowest OppA concentration that maintained a wide dynamic range of fluorescence during the melt phase was selected as the set protein concentration to be utilized for screening purposes to improve the sensitivity of the assay. This was determined to be 0.05 mg/mL for OppA1, OppA2, OppA3, and OppA4 and 0.075 mg/mL for OppA5.

The following was performed to optimize the concentration of tripeptide RFA for utilization as a positive control of OppA binding for high-throughput screening. A 10× solution of the appropriate OppA assay concentration and an 8× dye solution were prepared in assay buffer. Eight 20× RFA solutions spanning a range of 78 μM to 10 mM were prepared in assay buffer for a final assay concentration range of 3.9 to 500 μM. To each sample well was added 2 μL of 10× OppA, 2.5 μL 8× dye, 1 μL of respective 20× RFA solution, and 14.5 μL of assay buffer, for a total well volume of 20 uL. A control containing only OppA with dye in assay buffer was prepared as a reference sample. Two negative controls with respect to fluorescence were also prepared, one containing only dye in assay buffer and one containing RFA and dye in assay buffer. Four replicates were run. The TSA was run using Applied Biosystems Viia 7 Real-Time PCR System according to the Protein Thermal Shift™ Dye Kit manufacturer’s protocol. The resulting output file was then processed in Protein Thermal Shift™ Software version 1.3 to yield the *T*_m_ and Δ*T*_m_ for each sample calculated from a Boltzmann fit to the melt curve. The Z′-factor for the *T*_m_ of each RFA concentration was calculated using the following standard equation:


$$Z^{\prime }factor=1-\frac{3\left(\sigma_{r}+\sigma_{p}\right)}{\left|\mu_{p}-\mu_{r}\right|}$$

where *σ*_r_ and *σ*_p_ are the standard deviations of *T*_m_ of the reference and positive controls, respectively, and where *μ*_r_ and *μ*_p_ are the means of the *T*_m_ of the reference and positive controls, respectively. Concentrations of RFA that produced an OppA *T*_m_ shift yielding a Z′-factor >0.5 were selected as the designated RFA concentrations to be used in the high-throughput compound screen. The concentrations of RFA selected for use in TSA screening were 8 μM for OppA1 and OppA2 and 500 μM for OppA4 and OppA5. No concentration of RFA was found to shift the OppA3 *T*_m_.

### 2.6 Thermal shift assay for high-throughput screening of inhibitors against OppA2

A TSA screen of 2,240 compounds from a ChemBridge diversity library was tested for binding in singlet against OppA2 in a 384-well format. Compounds were supplied as 10-mM stock solutions in DMSO and were dispensed in volumes of 0.1 μL (200×) using a Beckman Coulter Echo 525 Liquid Handler. A volume of 0.1 μL DMSO was also dispensed into all designated reference, negative, and positive control wells for a final DMSO content less than 0.5% in each well. A Biotek Multiflo Dispenser (Agilent) was utilized to dispense 20 μL of solution containing 0.05 mg/mL OppA2 and 1X Protein Thermal Shift™ dye in assay buffer into all reference control and test compound wells for a final compound assay concentration of 50 μM. Additionally, 20 μL of solution containing 0.5 mg/mL OppA2, 8 μM RFA, and 1X dye was added to all positive control wells, and 20 μL of 1X dye was added to all negative control wells. The TSA was run using Applied Biosystems Viia 7 Real-Time PCR System according to the Protein Thermal Shift™ Dye Kit manufacturer’s protocol. The resulting output file was then processed in the Protein Thermal Shift™ Software version 1.3 for initial formatting, and the raw data was imported into GraphPad Prism 10 (GraphPad, USA) for analysis. All data were pruned to a range of 45°C to 75°C, and the baseline was removed by defining the baseline as the first value and subtracting that from all values. A nonlinear regression curve fit was selected, applying the Boltzmann sigmoidal as the model to fit to the melt curve. The *T*_m_ was defined as the V50 calculated from the Boltzmann fit. Compounds that produced OppA2 Δ*T*_m_ ≥ twice the standard deviation of the reference control *T*_m_ were classified as hits.

Hit validation was then carried out for select hits against OppA2 using the 10-mM compound stock solutions in DMSO from the screening library according to the screening conditions above, except that each compound was tested in triplicate. Additional negative controls containing 50 μM compound, 1X dye, and buffer were also included. Compounds that maintained hit status were purchased as dry powders and then re-evaluated in triplicate from freshly prepared 10 mM stock solutions in DMSO. Confirmed hits were counter-screened in triplicate against OppA1, OppA3, OppA4, and OppA5 using the conditions determined above. The data was processed in the Protein Thermal Shift™ Software version 1.3 for *T*_m_ and Δ*T*_m_ calculations. The hit threshold for each protein was defined as described above.

### 2.7 Phenol red proliferation assay

Initial screens for growth inhibition were conducted using a phenol red proliferation assay in a 96-well format. *B. burgdorferi* cultures were grown to mid-logarithmic stage of growth and diluted to 1 × 10^5^ spirochetes/mL. The culture was plated in 96-well plates with inhibitor or vehicle control in triplicate. The plates were incubated at 37°C, 5% CO_2_, and the plates were read by an AgileReader™ plate reader (ACTGene, Inc., USA) at OD_415_ daily. OD_415_ were plotted using PRISM 10 where an increase in OD_415_ correlates with an increase in spirochete growth as a function of media acidification. Micrographs were taken on an Axiolab 5 microscope with a 40× objective, and images were processed using ImageJ.

### 2.8 *B. burgdorferi* growth curves

Compounds identified in the growth inhibition screen were further assessed via *B. burgdorferi* growth curves. *B. burgdorferi* cultures were grown to mid-logarithmic stage of growth and diluted to 1 × 10^4^ spirochetes/mL. Growth curves were prepared in triplicate with inhibitor (150–50 mM) or vehicle control, and spirochetes were enumerated daily using darkfield microscopy. Spirochete concentrations were plotted using PRISM 10.

### 2.9 *E. coli* growth curves

Compounds found to have an inhibition activity against *B. burgdorferi* were tested against a lab strain of *E. coli.* An overnight culture of *E. coli* TOP10 in LB was diluted to OD_600_ = 0.70. The culture was plated in triplicate in a 96-well plate with inhibitor or vehicle control and incubated at 37°C, 220 rpm, and the plate was read by an AgileReader™ plate reader at OD_600_ hourly. OD_600_ was plotted using PRISM 10.

## 3 Results

### 3.1 Validation of peptide binding by thermal shift assay

**Figure 1:**
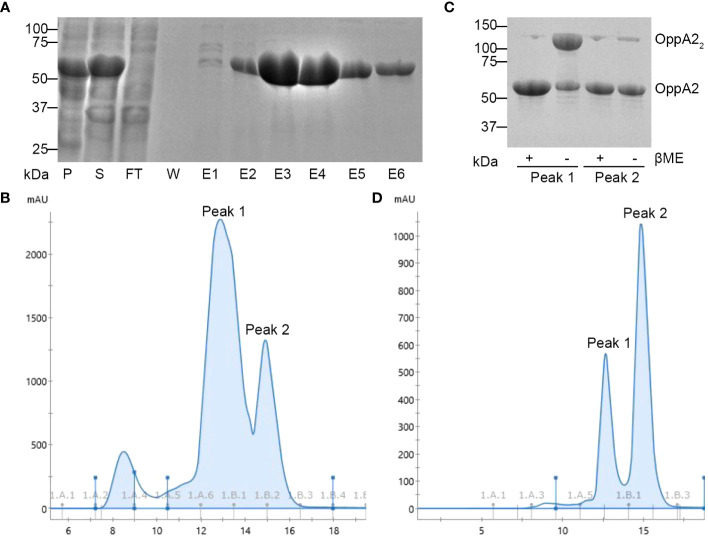
Analysis of purified recombinant OppA2. **(A)** Coomassie-stained SDS-PAGE gel of affinity-purified OppA2. P, pellet; S, soluble fraction; FT, flow through; W, wash; E, elution; protein ladder shown in kDa. **(B)** SEC profile of OppA2 without treatment; peak 1 and peak 2 represent the dimer and monomer composition, respectively. **(C)** Coomassie stained SDS-PAGE gel of SEC-purified OppA2 from either peak 1 or peak 2 with or without βME; protein ladder shown in kDa. **(D)** SEC profile of OppA2 after treatment with DTT; peak 1 and peak 2 represent dimer and monomer composition, respectively.

To develop a high-throughput screening tool for inhibitor identification, we first needed to validate a binding assay which would readily identify OppA binding. Jones et al. previously utilized a TSA to identify peptides bound by recombinant OppA from *Moraxella catarrhalis* (Jones et al., 2014). To this end, we generated a plasmid to express recombinant *B. burgdorferi* OppA2, our primary target for inhibitor screening, as described in “Materials and methods”. Recombinant protein was expressed without the N-terminal lipoprotein signal sequence, and the resultant protein included an N-terminal 6-His tag to aid in purification. Recombinant protein was first affinity-purified and then further purified and analyzed via size exclusion chromatography (SEC). While reducing SDS-PAGE analyses demonstrated a single band of appropriate size (60.74 kDa; **Figure 1A**), gel filtration chromatography showed two peaks that correlated to both monomeric and dimeric species (**Figure 1B**). Given that OppAs function as monomers, it was predicted that the N-terminal cysteine following the lipoprotein signal sequence cleavage site resulted in the formation of homodimers in solution. Protein from the two peaks produced during SEC was analyzed under reducing and non-reducing conditions by SDS-PAGE. The peak that correlated with dimeric species showed a larger protein band when not treated with a reducing agent, while treatment with β-mercaptoethanol resulted in a shift to a single, monomeric band (**Figure 1C**). When we treated affinity-purified protein with 1 mM DTT, SEC confirmed a shift to monomeric form (**Figure 1D**). We further confirmed proper folding of OppA2 by secondary structure analysis using microfluidic modulation spectroscopy (MMS; **Supplementary Figures S1A–C**). We had previously demonstrated that the homology models of *B. burgdorferi* OppAs against the OppA4 crystal structure provided robust models given the highly conserved secondary structures of the OppA (Groshong et al., 2017) and generated a homology model using the crystal structure of *B. burgdorferi* OppA4 (PDB:4GL8) (Fairman et al., 2012; Groshong et al., 2017). The percent higher order structure was determined for the homology model and compared to the MMS data (**Supplementary Figure S1D**). Indeed the secondary structure composition of purified, recombinant OppA2 was consistent with that of the OppA2 model, confirming native protein conformation (**Supplementary Figure S1D**).

**Figure 2:**
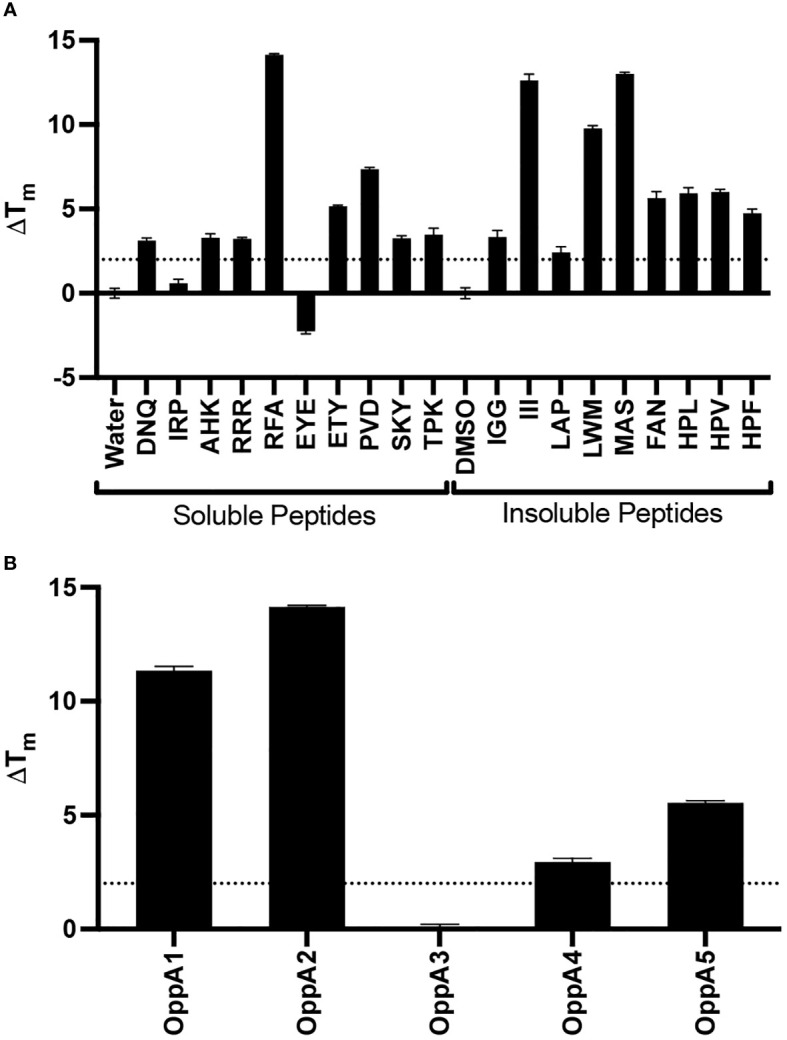
TSA results of tripeptide binding to OppAs. **(A)** Plot of Δ*T*_m_ for peptides against OppA2. **(B)** Plot of Δ*T*_m_ for RFA peptide against OppA1–5. The dotted line represents Δ*T*_m_ = 2°C threshold used to denote binding.

To determine whether a TSA would aid in the identification of compounds that bind OppA2, we tested our recombinant protein with a small peptide repertoire, including peptides that have previously been shown to bind OppA2 (Lin et al., 2001). We optimized the TSA protein and peptide concentrations (0.1 mg/mL protein and 1 mM peptide) and assessed the binding of these test peptides using a highly conservative Δ*T*_m_ ≥ 2°C as a readout for peptide binding (**Figure 2A**). Peptides previously demonstrated to bind OppA2 demonstrated Δ*T*_m_ ≥ 2°C shifts (Δ*T*_m_: HPL, +6.21; HPV, +6.28; HPF, +3.02). Furthermore, 14 other peptides tested showed Δ*T*_m_ ≥ 2°C, while two peptides shifted Δ*T*_m_ ≤ 2°C. Peptide RFA generated the highest Δ*T*_m_ for OppA2 and was tested against the other *B. burgdoferi* OppAs. OppA1, OppA3, OppA4, and OppA5 were expressed and purified using the same protocol as described above. RFA demonstrated binding via TSA to all other *B. burgdorferi* OppAs with the exception of OppA3 (**Figure 2B**). Overall, none of the peptides tested showed binding to OppA3 (data not shown). Wang et al. suggested that OppA3 binding was significantly affected by pH, with pH 2.2–5.0 representing optimum conditions (Wang et al., 2004). However, when we tested binding of all peptides at pH 5.0 for OppA3, there were no positive TSA results (data not shown). RFA was selected to serve as a positive control for the inhibitor screen given its strong thermal shift and promiscuity among the OppAs.

### 3.2 TSA screen and hit validation of potential inhibitors against OppA2

In preparation for running a high-throughput TSA screen of a Chembridge diversity library against OppA2, the ideal concentration of OppA2 that would conserve protein while maintaining a robust fluorescence signal was optimized and determined to be 0.05 mg/mL. To utilize RFA as a positive control during screening, various concentrations of RFA were screened against OppA2 to determine the lowest concentration of RFA that gave a desirable Z′-factor between 0.5 and 1, a value that describes a sufficiently wide signal-to-noise ratio in biochemical assays (Zhang et al., 1999). The concentration of RFA selected was 8 μM, which produced an OppA2 *T*_m_ shift yielding a Z′-factor of 0.82, indicating that this concentration could convey statistical confidence that the differences between positive and reference controls in the TSA are sufficient for hits to be reliably identified while minimizing false positives or negatives.

**Figure 3:**
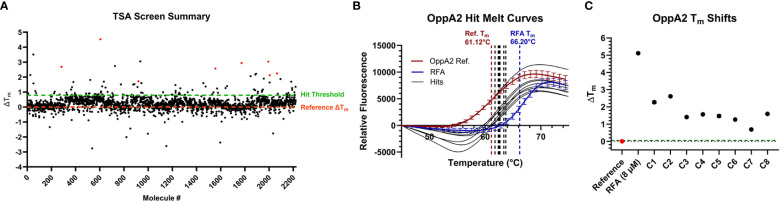
TSA results of inhibition screen. **(A)** Scatterplot of all Δ*T*_m_ for the 2,240 compounds screened against OppA2. The orange dashed line represents the Δ*T*_m_ of the OppA2 reference control. The green dashed line represents the average hit threshold across all plates screened. The red data points represent the eight validated hit compounds. **(B)** OppA2 melt curves from the final validation of hit compound solutions freshly prepared from dry powder. **(C)** Plot depicting the extent of Δ*T*_m_ of OppA2 in the presence of the validated hit compounds. The red data points represent the reference controls, the blue data points represent the positive control, and the green dashed line represents the Δ*T*_m_ hit threshold ≥ twice the *T*_m_ standard deviation of the reference control.

As a pilot screen, 2,240 compounds from the compound library containing 10-mM stock solutions in DMSO were screened by TSA at concentrations of 50 μM in 384-well plates using the optimized OppA2 and RFA assay concentrations (**Figure 3A**). All plates screened generated Z′-factors ranging from 0.58 to 0.78, yielding good statistical confidence. For each plate screened, the hit threshold was defined as greater than or equal to twice the standard deviation of the OppA2 reference control, a common best practice described for TSA screens (Mashalidis et al., 2013). With this criterion, 96 initial hits were identified. Due to the majority of hits producing Δ*T*_m_s slightly above the hit threshold (**Figure 3A**), we chose to carry forward the top 20 hits displaying the greatest Δ*T*_m_s for hit validation in triplicate from the library solutions. From the hits validated, eight commercially available compounds were purchased as dry powders for the preparation of fresh DMSO stock solutions for an additional round of hit validation as well as for antimicrobial testing. All eight compounds maintained hit status against OppA2 (**Figures 3B, C**; **Table 3**).

**Table 3:** Compound table with TSA summary.

Ligand	Structure	Δ*T*_m_				
		OppA1	OppA2	OppA3	OppA4	OppA5
**RFA**	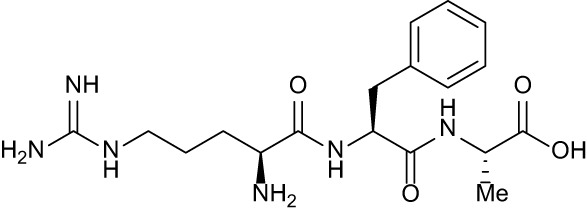	+ 3.81	+ 5.12	Not Available	+ 1.58	+ 2.48
**C1**	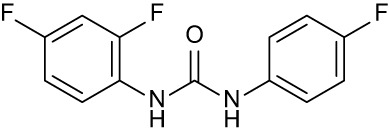	+ 2.36	+ 2.27	−0.16	+ 1.22	+ 0.86
**C2**	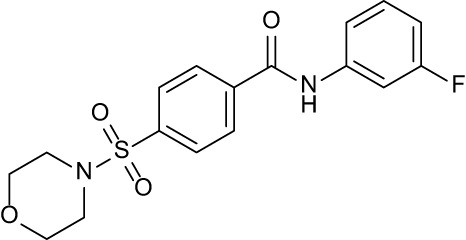	+ 2.84	+ 2.61	+ 0.07	−0.56	+ 0.02
**C3**	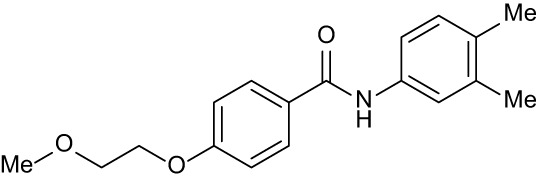	+ 1.90	+ 1.41	+ 1.64	+ 1.17	+ 0.31
**C4**	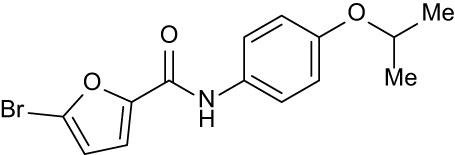	+ 2.86	+ 1.57	−0.44	−0.53	+ 0.66
**C5**	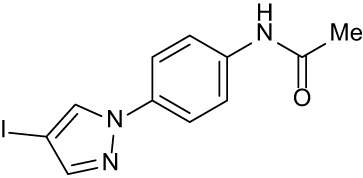	+ 2.60	+ 1.47	+ 1.13	+ 2.10	+ 0.81
**C6**	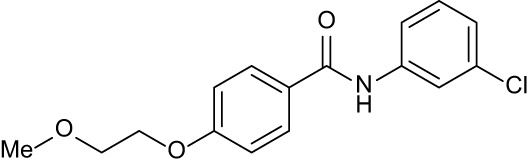	+ 2.47	+ 1.27	+ 0.36	+ 1.32	+ 0.91
**C7**	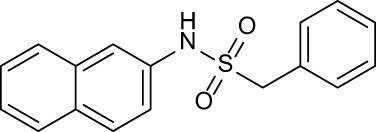	+ 1.26	+ 0.69	−0.02	+ 0.32	−0.08
**C8**	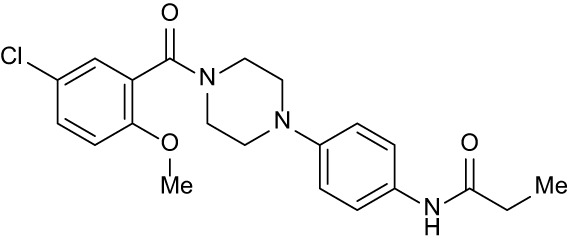	+ 2.27	+ 1.59	+ 0.43	+ 0.71	+ 0.22

Green numbers represent positive hits. Red numbers indicate that the compound failed to produce ΔT_m_ above the hit threshold.

### 3.3 Effects of inhibitors on *B. burgdorferi* growth

**Figure 4:**
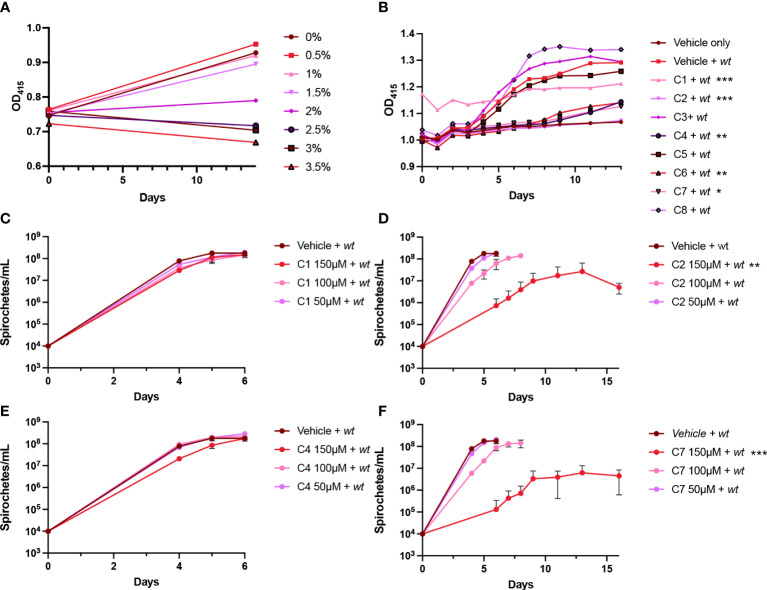
Effects of hit compounds on *Borrelia burgdorferi* growth. **(A, B)** Phenol red proliferation assay demonstrating the inhibition of DMSO **(A)** and hit compounds **(B)** on *B. burgdorferi* growth by reading OD_415_. **(C–F)** Growth curves showing the effects of C1, C2, C4, and C7, respectively, at varying concentrations (150, 100, and 50 μM) on *B. burgdorferi* as measured by spirochete enumeration by darkfield microscopy. The dotted line denotes the limit of detection by microscopy. All samples were evaluated in triplicate; error bars represent SEM. *p< 0.05, **p< 0.01, ***p< 0.005 compared to the vehicle control using unpaired *t* test. Statistics for C1 in the phenol red assay was determined by utilizing a baseline correction.

To determine the effects of the eight hit compounds identified by TSA on *B. burgdorferi* growth, we developed a high-throughput bacterial growth screen. The BSK-II growth medium contains phenol red, providing a distinct red color at initial pH for spirochete cultivation (pH 7.5). During bacterial cultivation, cellular respiration releases acidic byproducts into the media, changing the color of the indicator to yellow. We utilized the presence of phenol red in the media to develop a phenol red proliferation assay for *B. burgdoferi* where absorbance at 415 nm (OD_415_, absorbance maxima at acidic conditions) measures the yellow spectra and increased OD_415_ readings directly correlate with increased cell proliferation. As inhibitors were dissolved at 10 mM in 100% DMSO, we first tested the maximum percent DMSO that could be added to *B. burgdorferi* culture without significantly impacting growth (**Figure 4A**). We found that the addition of more than 1.5% DMSO resulted in the delayed growth of the spirochete culture. Therefore, we began our inhibitor growth screen at the maximum concentration allowable based on DMSO tolerance (1.5% DMSO or 150 μM inhibitor). Inhibitors or vehicle controls were added to *B. burgdorferi* strain B31 cultures in triplicate in a 96-well format by reading OD_415_ daily for 13 days (**Figure 4B**). Addition of C1 resulted in a shift of OD_415_ at *T* = 0, perhaps due to competing absorbance at OD_415_, but failed to show an increase in OD_415_ during cultivation, suggesting delayed growth. Addition of other compounds had no effect on OD_415_ at initial timepoints and only C2, C4, and C7 failed to demonstrate an increase in OD_415_ that denoted robust growth. Darkfield microscopy images confirmed lower bacterial concentrations for these samples (**Supplementary Figure S2**).

The four compounds that displayed growth defects in the phenol red proliferation assay were subsequently evaluated by *B. burgdorferi* growth curves and evaluated for minimal inhibitory concentrations (MIC; **Figures 4C–F**). Cultures of *B. burgdorferi* strain B31 were prepared in triplicate, and C1, C2, C4, and C7 at 150, 100, or 50 μM or vehicle control were added. The growth curves demonstrated almost no effect from C1 at any concentration and a slight inhibition from C4 at 150 μM. Alternatively, C2 and C7 displayed a significant inhibition of growth at 150 μM and a slight inhibition at 100 μM, while 50 μM had no impact, suggesting that the maximum concentration is close to the compound MIC. However, increasing the compounds above 150 μM would have resulted in an adverse growth effect due to DMSO concentration; therefore, we could not evaluate the impact of higher concentrations of these inhibitors during this study.

### 3.4 Inhibitors and their effect on other OppAs

**Figure 5:**
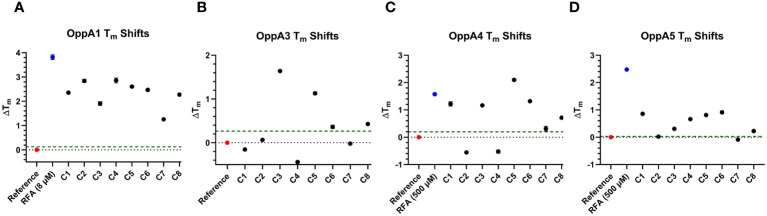
TSA results for other OppAs. **(A–D)** Plots depicting the extent of Δ*T*_m_ of OppA1, OppA3, OppA4, and OppA5, respectively, in the presence of the validated OppA2 hit compounds. The red data points represent the reference controls, the blue data points represent the positive controls (none for OppA3), and the green dashed line represents the Δ*T*_m_ hit threshold ≥ twice the *T*_m_ standard deviation of the reference control.

The highly conserved structure of OppAs provides the possibility for inhibitors to target multiple OppAs. To evaluate whether the test compounds displayed binding to other *B. burgdorferi* OppAs, we repeated the inhibitor TSA with OppA1, OppA3, OppA4, and OppA5 (**Figures 5A–D**). The same process was employed to identify the ideal TSA concentrations of OppAs as was described for OppA2 screening. After determining the appropriate assay concentrations for OppA1 (0.05 mg/mL), OppA3 (0.05 mg/mL), OppA4 (0.05 mg/mL), and OppA5 (0.075 mg/mL), the concentration of RFA was optimized, as described, for each OppA to generate a strong Z′-factor for the TSA screening of compounds. The best RFA concentration for use with OppA1 was determined to be 8 μM (Z′-factor of 0.62) and with OppA4 and OppA5 to be 500 μM (Z′-factors of 0.51 and 0.53, respectively). OppA3 *T*_m_ failed to be shifted by RFA or any other tripeptides initially screened, consistent with initial peptide screens for OppA3 (**Figure 2B**) and, therefore, lacked a positive control in the TSA evaluation of hit compounds. The eight OppA2 hit compounds were then tested at 50 μM in triplicate against OppA1, OppA3, OppA4, and OppA5, with hit status defined as producing a Δ*T*_m_ of greater than or equal to twice the standard deviation of the reference control (**Figures 5A–D**, **Table 3**). The level of this threshold varied between OppAs according to the extent of *T*_m_ variation across reference control replicates. All eight compounds achieved hit status against OppA1, an interesting finding considering that the binding cavities of OppA1 and OppA2 are characterized by opposite electrostatic distributions, according to past homology modeling (Groshong et al., 2017). C3, C5, C6, and C8 achieved hit status against OppA3, although it should be noted that due to lack of a positive control in the OppA3 TSA, the data should be interpreted with discernment. All compounds except C2 and C4 were identified as hits against OppA4. Finally, all compounds except C2 and C7 were identified as hits against OppA5, which is peculiar considering that C2 and C7 were the only two compounds that successfully inhibited the growth of *B. burgdorferi*. Assuming that the OppA5 TSA results indicate lack of binding of C2 and C7 to OppA5, then it is likely that the antimicrobial effects of C2 and C7 on *B. burgdorferi* are not conveyed through OppA5 binding. Overall, TSA showed that C2 and C7, the two compounds responsible for inhibiting *B. burgdorferi* growth, primarily interact with OppA1 and OppA2, with C7 barely achieving hit status against OppA4.

### 3.5 Evaluation of inhibitors against *oppA2tn* mutant

**Figure 6:**
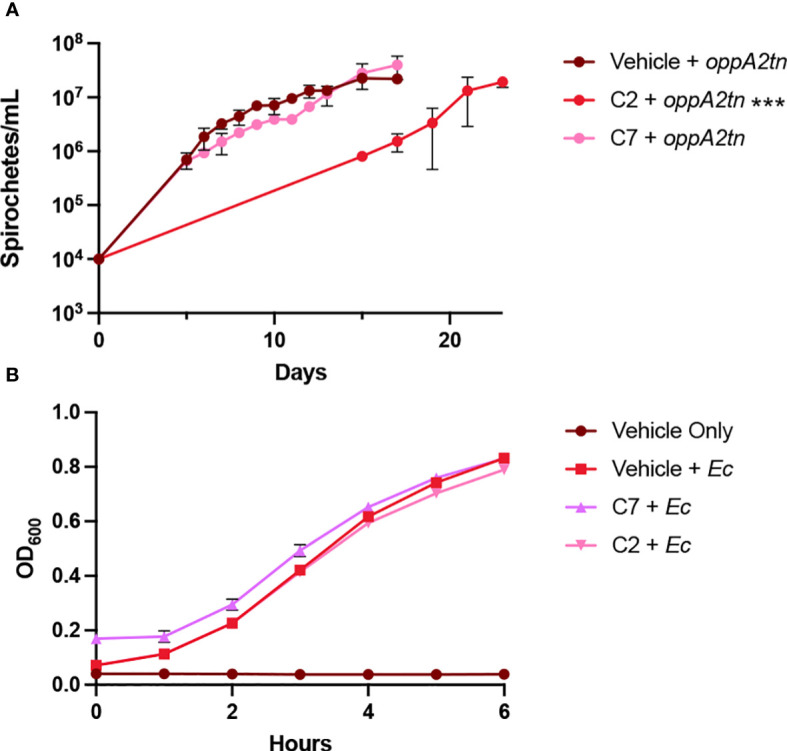
Specificity of hit compounds. **(A)** Growth curves showing the effects of C2 and C7 *B. burgdorferi oppA2tn* mutant as measured by spirochete enumeration by darkfield microscopy. The dotted line denotes the limit of detection by microscopy. **(B)** Impact of C2 and C7 on *E. coli* growth as measured by OD_600_. All samples were evaluated in triplicate; the error bars represent SEM. ***p< 0.005 compared to vehicle control using unpaired *t* test.

To begin to probe a possible mechanism of inhibition, we also tested inhibitor effects on a strain of *B. burgdorferi* with an insertional mutation in *oppA2* (*oppA2tn*) to determine whether the loss of OppA2 impacts the inhibitors’ effects. Growth curves were repeated with *oppA2tn* and 150 μM inhibitor or vehicle control (**Figure 6A**) Interestingly, C7 had little effect on *oppA2tn* growth compared to the vehicle control. Alternatively, C2 significantly impacted growth of the mutant strain. The varying impact of these compounds on the *oppA2tn* mutant suggests a difference in OppA targeting or compound activity.

### 3.6 Evaluation of inhibitors against *E. coli*

To determine whether the inhibition activity was specific to *B. burgdorferi*, we evaluated the impact of C2 and C7 on *E. coli* growth. Unlike *B. burgdorferi*, the Opp system in *E. coli* is an accessory nutrient uptake system and is not required for growth. *E. coli* TOP10 lab strain was used to conduct a growth curve with 150 μM inhibitor or vehicle control by recording OD_600_ hourly (**Figure 6B**). Neither C2 nor C7 displayed growth inhibition against *E. coli* in comparison to the vehicle control, suggesting that these inhibitors may display inhibition only when peptide transport is critical for growth.

## 4 Discussion

Studies demonstrating that the Opp system of *B. burgdorferi* is required for viability exposed a unique and selective target for inhibitor development. Most bacteria initiate a stringent response system [i.e., (p)ppGpp/Rel] to modulate bacterial growth in response to amino acid starvation, allowing the bacteria to survive until nutrients are restored (Wang et al., 2020). The maladaptive response of *B. burgdorferi* to amino acid starvation as demonstrated by ablation of the Opp system suggests an uncoupling of amino acid stress from the stringent response system (Groshong et al., 2017). Without a rescue system in place, *B. burgdorferi*’s reliance on peptide uptake for amino acids exposes a significant liability of the spirochete’s unique physiology. While the bacterium likely does not encounter a peptide-replete environment during active replication either in the tick or the mammal, disruption of OppA function via pharmacological intervention would render the bacterium unable to obtain critical physiological building blocks during critical growth phases. Due to *B. burgdorferi*’s unusual dependence on peptide transport, we evaluated the potential to target this system with small molecule inhibitors.

After demonstrating that TSA could be employed to identify peptides that bind OppAs in agreement with previous transport studies, we screened 2,240 compounds in a high-throughput TSA. We reasoned that, regardless of inhibitory action, a candidate compound would be required to bind an OppA to elicit an effect. For the eight hit compounds identified by TSA against OppA2, we developed the phenol red proliferation assay as a high-throughput growth screen. As *B. burgdorferi* requires daily enumeration via darkfield microscopy to track the bacterial growth, this assay provided a preliminary screening mechanism prior to performing traditional *B. burgdorferi* growth curves. Indeed the phenol red proliferation assay identified four out of the eight compounds that had some effect on growth. Of these four compounds, C1 and C4 had minimal inhibition of growth, while C2 and C7 significantly inhibited *B. burgdorferi* using our standard growth curve analysis. Subsequently, when we evaluated the ability of C2 and C7 to bind other OppAs, we found that C2 exhibited higher Δ*T*_m_s for OppA1 and OppA2 (+2.84 and +2.61, respectively), while C7 exhibited lower Δ*T*_m_s (+1.26 and + 0.69, respectively).

The potential activity for these compounds could result from the ability to bind OppAs in a way that prevents peptide transport. This would include occlusion of the binding site or locking into the hinge region. Alternatively, the compounds may have intracellular targets that impact bacterial growth, only binding an OppA for transport into the cytoplasm. When *oppA2tn*, a strain missing OppA2, was treated with these compounds, C2 did inhibit growth, while C7 lacked an effect on growth compared to the control. If the activity of these compounds results from inactivating OppAs through binding, the maintained efficacy of C2 against *oppA2tn* may be explained by the high Δ*T*_m_ shifts exhibited by C2 against both OppA1 and OppA2, allowing it to maintain significant growth inhibition against *oppA2tn* because of its additional activity against OppA1. This would be consistent with OppA1 and OppA2 being the primary OppAs responsible for bulk peptide transport in *B. burgdorferi*, a theory that is buttressed by the genetic conservation of only OppA1 and OppA2 in the closely related relapsing fever species of *Borrelia.* The lack of C7-mediated growth inhibition in *oppA2tn* may be due to the relatively low Δ*T*_m_ shifts against OppA1 and OppA2, such that its activity against OppA1 is not significant enough to further stunt growth in *oppA2tn*. If compound inhibition resulted from activity once transported into the cell, strong binding of C2 to OppA1 may maintain effective transport in the absence of OppA2 to inhibit growth. On the other hand, C7 may have lost inhibition with the loss of OppA2 for transport given the lower Δ*T*_m_ shifts and the fact that 150 μM appears to be the MIC threshold. It should be noted that some compounds identified as binding multiple OppAs by TSA (i.e., C5) displayed no inhibitory effects on *B. burgdorferi*. Moreover, while *E. coli* would not display inhibition at the loss of OppA function, it is possible that the compounds did not bind *E. coli* OppA and, therefore, were not transported or that the compound is not inhibitory once transported into the *E. coli* cytoplasm. Unfortunately, the solubility of the two candidate compounds prevented additional investigations of compound activity such as identification of binding sites via crystallography and binding affinities through ITC.

Both C2 and C7 were analyzed by ADMETlab3.0 to evaluate their predicted pharmacokinetic properties and likelihood as drugs (Dong et al., 2018). In brief, both compounds meet the Lipinski Rule of 5 for drug-likeness, with C7 having the lower molecular weight and more room for lead optimization. Both compounds are predicted to have high Caco-2 permeability and oral bioavailability of 50% or greater, which are desirable properties for potential oral agents. C7 is predicted to be relatively stable to human liver microsomes (HLM) with a half-life (*t*_1/2_) greater than 30 min, whereas C2 is predicted to have low HLM stability with a *t*_1/2_ less than 30 min. However, metabolic stability is often a factor that can be addressed during lead optimization to overcome those liabilities. C2 and C7 are predicted to have short *in vivo t*_1/2_ in plasma with values of 0.68 and 1.18 h, respectively. Due to the simplicity of the Chembridge diversity library, many of the compounds are meant to serve as initial hit scaffolds for downstream optimization. Therefore, improving metabolic stability may need to be a priority for these compounds in the future if they are to be taken into efficacy assays *in vivo*. Meanwhile, it was encouraging to see promising initial Caco-2 and bioavailability predictions for these scaffolds, indicating a potential for oral dosing.

Overall, we have initiated a drug discovery pipeline that takes advantage of a previously unexplored and unique bacterial target. One caveat to these *in vitro* studies is that the cultivation of *B. burgdorferi* in BSK-II at 37°C does not faithfully replicate the mammalian host environment as c-di-GMP is being generated during *in vitro* growth and RpoS expression is only achieved during high-density growth (Caimano et al., 2019; Groshong et al., 2021a). Alternatively, room temperature cultivation to replicate unfed tick conditions is also complicated by the incredibly nutrient-rich nature of BSK-II which provides signals to grow *in vitro*, while spirochetes are mostly dormant during tick molt. Overall, OppA inhibition is likely to be most effective during metabolically active stages such as tick engorgement (OppA1–4 active) or proliferation within the mammal (primarily OppA2 and OppA5 active) (Groshong et al., 2017). Continued compound screening will help to identify common structural features of inhibitory compounds to further inform compound design and testing for the optimization of antimicrobial efficacy. Moreover, such molecules will help further elucidate the role that the Opp system proteins have on growth of *B. burgdorferi* and allow progression into *in vivo* model testing.

## Data availability statement

The original contributions presented in the study are included in the article/**Supplementary Material**. Further inquiries can be directed to the corresponding authors.

## Author contributions

KH: Data curation, Formal analysis, Investigation, Validation, Visualization, Writing – original draft, Writing – review & editing. AK: Investigation, Methodology, Validation, Visualization, Writing – original draft, Writing – review & editing. DF: Conceptualization, Data curation, Formal analysis, Funding acquisition, Investigation, Methodology, Resources, Supervision, Writing – original draft, Writing – review & editing. AG: Conceptualization, Data curation, Formal analysis, Funding acquisition, Investigation, Methodology, Resources, Supervision, Validation, Visualization, Writing – original draft, Writing – review & editing.
