# Supplementary material for: Unguarded liabilities: Borrelia burgdorferi’s complex amino acid dependence exposes unique avenues of inhibition
Source: Front Antibiot. 2024 May 20;3:1395425. doi: 10.3389/frabi.2024.1395425 (PMC11732028; doi:10.3389/frabi.2024.1395425)
Supplement: Supplementary file 1 [file DataSheet_1.pdf]

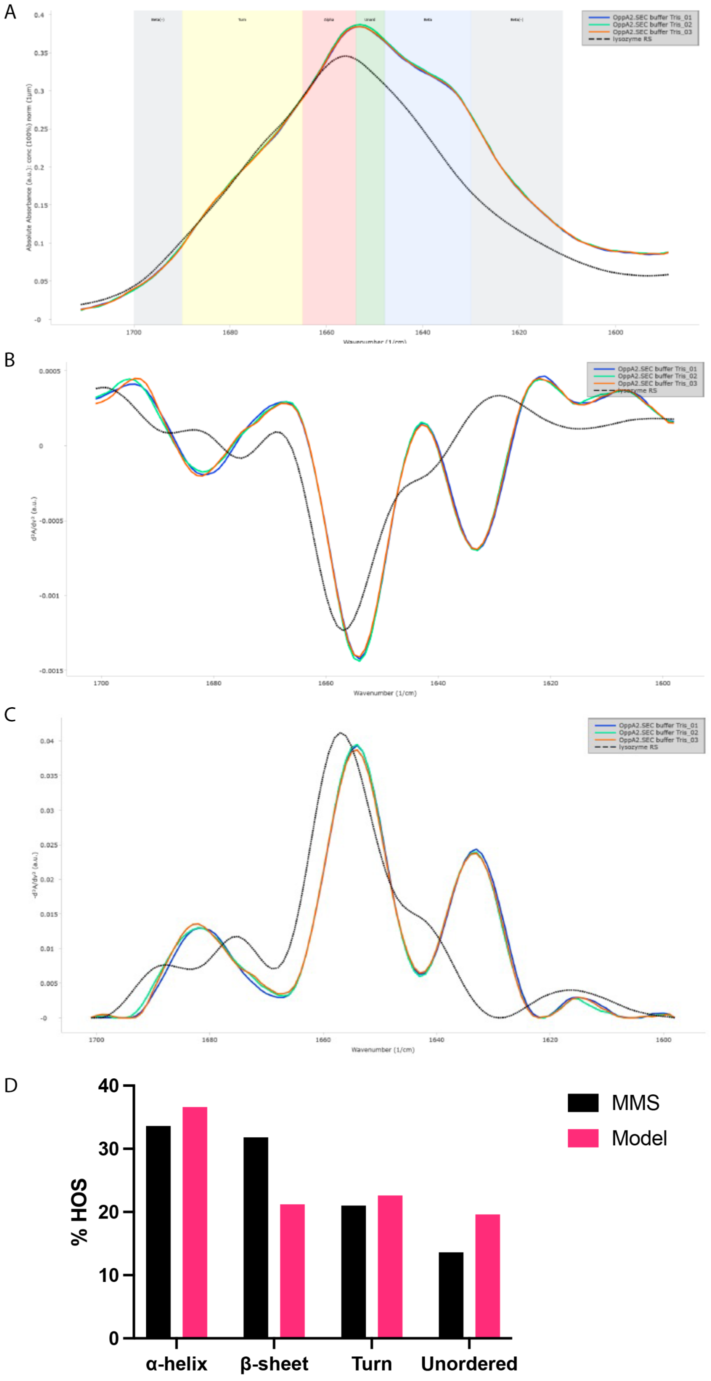

Supplementary Figure 1. (A-D) Secondary structure analysis by MMS for OppA2. (A) Absolute absorbance spectum (B) Second derivative of absolute absorbance spectra, (C) Second derivative inverted and baselined. OppA2 was run in triplicate shown by blue, green, and red lines, with respect to model protein (lysozyme) shown in black. (D) Comparison of %HOS from MMS and homology model of OppA2 as calculated by STRIDE server.

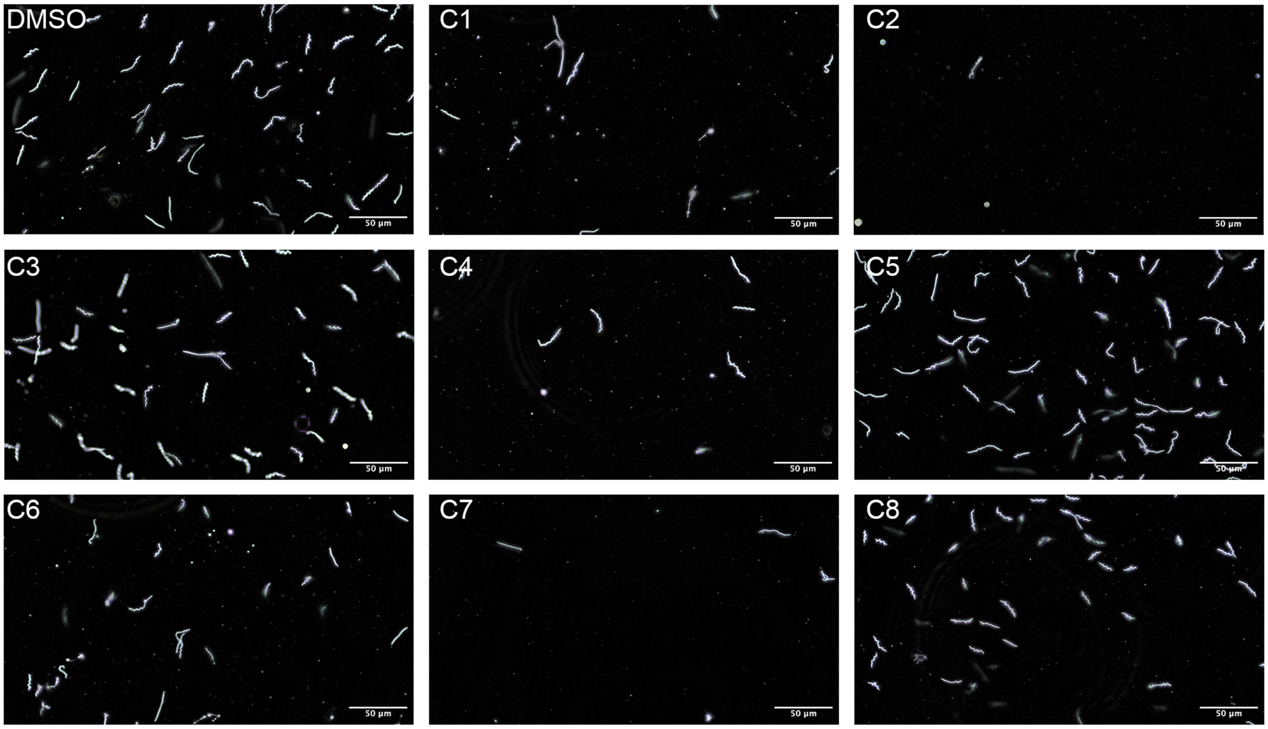

Supplementary Figure 2. Darkfield microscopy of samples from Figure 4B. Images are were taken with a 40x objective and scale bars are 50µm.
